# Clinical predictors of prognosis in patients with traumatic brain injury combined with extracranial trauma

**DOI:** 10.7150/ijms.54913

**Published:** 2021-02-05

**Authors:** Chengli Liu, Jie Xie, Xinshuang Xiao, Tianyu Li, Hui Li, Xiangjun Bai, Zhanfei Li, Wei Wang

**Affiliations:** Department of Traumatic Surgery, Tongji Hospital, Tongji Medical College, Huazhong University of Science and Technology, Wuhan 430030, P.R. China.

**Keywords:** prognosis, traumatic brain injury, multiple trauma, prognostic factor

## Abstract

**Objective:** The purpose of this study was to investigate whether routine blood tests on admission and clinical characteristics can predict prognosis in patients with traumatic brain injury (TBI) combined with extracranial trauma.

**Methods:** Clinical data of 182 patients with TBI combined with extracranial trauma from April 2018 to December 2019 were retrospectively collected and analyzed. Based on GOSE score one month after discharge, the patients were divided into a favorable group (GOSE 1-4) and unfavorable group (GOSE 5-8). Routine blood tests on admission and clinical characteristics were recorded.

**Results:** Overall, there were 48 (26.4%) patients with unfavorable outcome and 134 (73.6%) patients with favorable outcome. Based on multivariate analysis, independent risk factors associated with unfavorable outcome were age (odds ratio [OR], 1.070; 95% confidence interval [CI], 1.018-1.124; *p<*0.01), admission Glasgow Coma Scale (GCS) score (OR, 0.807; 95% CI, 0.675-0.965; *p<*0.05), heart rate (OR, 1.035; 95% CI, 1.004-1.067; *p<*0.05), platelets count (OR, 0.982; 95% CI, 0.967-0.997; *p<*0.05), and tracheotomy (OR, 15.201; 95% CI, 4.121-56.078; *p<*0.001). Areas under the curve (AUC) of age, admission GCS, heart rate, tracheotomy, and platelets count were 0.678 (95% CI, 0.584-0.771), 0.799 (95% CI, 0.723-0.875), 0.652 (95% CI, 0.553-0.751), 0.776 (95% CI, 0.692-0.859), and 0.688 (95% CI, 0.606-0.770), respectively.

**Conclusions:** Age, admission GCS score, heart rate, tracheotomy, and platelets count can be recognized as independent predictors of clinical prognosis in patients with severe TBI combined with extracranial trauma.

## Introduction

Traumatic brain injury (TBI) is the leading cause of mortality, long-term disability, and cognitive impairment that particularly affects young people worldwide 1, 2. Prognosis of patients with traumatic brain injury is still unclear, and multiple studies have tried to identify predictors of outcome in these patients. Some studies in TBI patients have shown that old age, low admission Glasgow Coma Scale (GCS) score, high head abbreviated injury scale (AIS) score, poor pupillary reflex, hypoxia, increased intracranial pressure and tracheotomy were related with poor functional outcome 3-7. In addition, high white blood cells, high neutrophil lymphocyte ratio (NLR), anemia, high blood glucose, high sodium, and high uric acid in routine blood test were closely related to poor prognosis of neurological function 8-12. Concentration of certain blood biomarkers, including S100B, glial fibrillary acidic protein (GFAP), tau, neuron-specific enolase (NSE), periostin, translocator protein, interleukin (IL)-8/33 and TWEAK, have also been reported as predictors of the final outcome 13-20.

Multiple trauma was defined as the injury associated with more than one body cavity or organ, which was considered to be the major cause of death and morbidity 21. As showed in previous clinical studies, TBI was often accompanied by injuries of extracranial sites 22. In patients with multiple trauma, prognostic factors included age, sex, ISS score, GCS score, injury mechanism, and systolic blood pressure 23-25. Condition of TBI patients with multiple injuries involves multiple parts, which are often severe and difficult to diagnose and treat, and may influence their prognosis. Hypotension and coagulopathy resulting from an extracranial injury were important factors for secondary injury to TBI 26. The above predictive factors should be evaluated thoroughly in patients with TBI and extracranial trauma.

Therefore, to study the influence of extracranial trauma on the prognosis of TBI, we analyzed patients with TBI combined with extracranial trauma and evaluated whether blood analysis at the admission and clinical characteristics can be used to predict the patients' prognosis.

## Material and methods

This retrospective study included patients with traumatic brain injury combined with extracranial trauma who were admitted to our department between April 2018 and December 2019 in Tongji Hospital. The study was approved by the local Ethics Committee. The diagnosis was based on a history of injury, clinical manifestations, and radiological examination. The inclusion criteria were as follows: (1) traumatic brain injury with head AIS ≥ 3, (2) at least one other body region with AIS ≥ 2, (3) admission Injury Severity Score (ISS) > 15, (4) interval from injury to hospital admission < 24 h, and (5) age ≥ 18 years. Patients with missing or incomplete data and isolated TBI were excluded.

Data regarding age, gender, injury mechanism, time interval from injury to admission, blood pressure, heart rate, pupil size, pupillary reflex, admission GCS score, ISS score, head AIS score, injury position, use of tracheal intubation and tracheotomy, and complications during hospitalization were collected from electronic medical records in Tongji Hospital. Patients were followed up and evaluated for Glasgow Outcome Scale-extended (GOSE) score 1 month after discharge.

Patients with TBI were diagnosed using head computed tomography (CT) at admission. Acute brain injuries assessed by CT imaging included the following: epidural hematoma (EDH), subdural hematoma (SDH), subarachnoid hemorrhage (SAH), cerebral hemorrhage, and skull fracture. The head abbreviated injury score (AIS) was an internationally recognized traumatic injury scoring system, including codes in 9 different regions: head, face, neck, thorax, abdomen, spine, upper extremity, lower extremity, whole body, and other 27. AIS was assessed as follows: minor (1 point), moderate (2 points), severe, not life-threatening (3 points), severe, life-threatening (4 points), critical (5 points), and lethal (6 points) 27-29. Thereby, AIS ≥ 3 was recognized as a severe TBI. The ISS score was calculated based on the severity of the highest AIS in up to three anatomic areas. ISS > 15 was used to define severe multiple trauma because it may predict 10% mortality rate of trauma patients 30. The GCS scores were classified as mild (13-15 points), moderate (9-12 points), and severe (3-8 points). The GOSE scores were dichotomized to two groups: favorable (good recovery to moderate disability; GOSE 5-8) and unfavorable (severe disability to death; GOSE 1-4) 31.

### Statistical analysis

Continuous data were expressed as mean ± standard deviation (SD), and compared using Student's *t* test. Categorical variables were analyzed by Pearson Chi-square or Fisher's exact probability test. To identify predictors independently associated with the prognosis, we performed multivariate logistic regression analysis with variables that had shown a statistical trend (*p* < 0.1) in univariate analysis. The corresponding 95% confidence interval (95% CI) was used to calculate and represent the odds ratio (ORs). The receiver operating characteristic (ROC) curve was used to show sensitivity and specificity of independent predictors to calculate the optimal cutoff points. The area under the ROC curve (AUC) was also calculated to judge the existence of discriminative ability. AUC > 0.5 indicated discriminative ability, AUC = 1 indicated complete discriminative ability, while AUC < 0.5 indicated no discriminative ability. All tests were two-sided, and *p* < 0.05 was considered statistically significant. Statistical analysis was conducted using SPSS 22.0.

## Results

**Figure 1:**
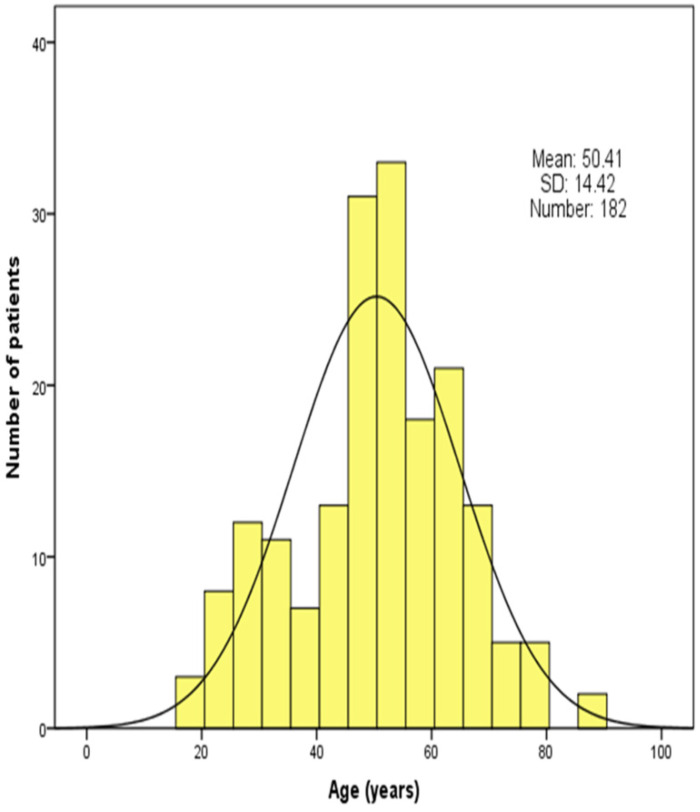
Age distribution of patients in the study population.

In total, 534 patients with traumatic brain injury were collected and assessed in this study. Among 182 patients finally included in the present study, unfavorable and favorable groups comprised 48 (26.4%) and 134 (73.6%) patients, respectively. Age distribution of the patients is shown in **Figure 1**. The mean age was 50.41 ± 14.42 years (range 18-84 years). There were 27 (14.8%) older patients (>64 years) with 15 (11.2%) patients in favorable groups and 12 (25.0%) in unfavorable groups, which were statistically different.

There were 135 (74.2%) men and 47 (25.8%) women in the study population. The mean time interval from the head injury to admission was 10.17±5.77 hours. The injury was mainly caused by traffic accident (118, 64.8%), followed by falls (43, 23.6%), assaults (5, 2.7%), and others/unknown (16, 8.8%). The admission GCS score was 11.68 ± 3.98 and admission ISS score was 26.17 ± 7.45, in which the head AIS score was 3.63 ± 0.67. Overall, 140 (76.9%) patients also had a chest injury, 64 (35.2%) had abdominal injury, while pelvic injury and limb injury were recorded in 27 (14.8%) and 79 (43.4%) patients, respectively. Based on the results of CT imaging, all patients had an abnormal CT brain scan and there were 69 (37.9%) patients with EDH, 111 (61.0%) with SDH, 125 (68.7%) with SAH, and 76 (41.8%) with intracranial hemorrhage. Skull fracture was present in 109 (59.9%) patients. A total of 32 (17.6%) patients were subjected to craniotomy and decompressive craniectomy. 60 (33%) and 55 (30.2%) patients underwent tracheal intubation and tracheotomy, respectively. During hospitalization, 31 (17%) patients developed pulmonary infection, and lower extremity venous thrombosis (LEVT) occurred in 30 (16.5%) patients. Total mortality at 1 month after discharge was 25 (13.7%). Baseline clinical characteristic of the two groups are displayed in **Table 1**.

White blood cell (WBC) count was 12.99 ± 4.33 (×10^9^/L), among which neutrophils count was 11.43 ± 4.09 (×10^9^/L) and lymphocytes count was 0.80 ± 0.51 (×10^9^/L). High WBC (>10×10^9^/L) was found in 138 (75.8%) patients, high neutrophils percentage (>75%) was recorded in 175 (96.2%) patients, whereas low lymphocytes percentage (<20%) was found in 178 (97.8%) patients. The NLR was 17.85 ± 10.44. Hemoglobin level was 110.90 ± 25.36 (g/L). Platelets count was 154.99 ± 53.83 (×10^9^/L). Low platelets (< 120×10^9^/L) and anemia (< 110 g/L) were found in 54 (29.7%) and 74 (40.7%) patients, respectively. Albumin was 35.43 ± 7.14 (g/L), and low albumin (< 35 g/L) was found in 78 (42.9%) patients. Blood Na+ concentration was 141.08 ± 3.57 (mmol/L), and high Na+ concentration (> 145 mmol/L) was present in 22 (12.1%) patients. Blood K+ concentration was 4.21 ± 0.53 (mmol/L), and low K+ concentration (< 3.5 mmol/L) was found in eight patients (4.4%). Blood urea nitrogen (BUN) was 5.82 ± 2.73 (mmol/L), and high BUN (>8 mmol/L) was noted in 16 (8.8%) patients. Blood creatinine was 78.96 ± 68.26 (µmol/L), and high creatinine (> 110 µmol/L) was recorded in 12 (6.6%) patients. Blood uric acid (UA) was 300.12 ± 100.48 (µmol/L), and high UA (> 417 µmol/L) was present in 26 (14.3%) patients. Blood glucose was 8.32 ± 2.78 (mmol/L), and hyperglycemia (> 8.0 mmol/L) was verified in 87 (47.8%) patients. For the overall results of coagulation tests, prothrombin time (PT) was 15.64 ± 2.78 (s), international normalized ratio (INR) was 1.60 ± 4.59, fibrinogen was 2.35 ± 1.01 (g/L), activated partial thromboplastin time (APTT) was 37.45 ± 7.44 (s), and thrombin time (TT) was 16.15 ± 2.01 (s). High PT (≥ 15s) was found in 96 (52.7%), high INR (> 1.2) in 79 (43.4%), low fibrinogen (< 2 g/L) in 65 (35.7%), high APTT (> 45s) in 16 (8.8%), and high TT (> 19s) in 13 (7.1%) patients. Laboratory parameters are shown in **Table 2.**

Univariate analysis showed that patients with favorable and unfavorable outcomes significantly differed in age, proportion of old men, admission GCS score, pupil size, pupillary reflex, heart rate, admission ISS score, head AIS score, skull fracture, craniotomy, tracheal intubation, tracheotomy, pulmonary infection, mortality, hemoglobin, platelets count, albumin, blood Na+, blood glucose level, PT, and APTT.

After adjusting for confound factors in the multivariate logistic model, age, admission GCS score, heart rate, tracheotomy, and platelets count were significant predictors of the 1-month outcome after discharge (**Table 3**).

The results indicated that among continuous variables, age (OR, 1.070; 95% confidence interval [CI], 1.018-1.124; *p*<0.01), admission GCS score (OR, 0.807; 95% CI, 0.675-0.965; *p*<0.05), heart rate (OR, 1.035; 95% CI, 1.004-1.067; *p*<0.05), and platelets count (OR, 0.982; 95% CI, 0.967-0.997; *p*<0.05) were independently associated with the unfavorable outcome at 1 month after discharge. The results also showed that among categorical variables tracheotomy (OR, 15.201; 95% CI, 4.121-56.078; *p*<0.001) was independently related with the unfavorable outcome at 1 month after discharge.

**Figure 2:**
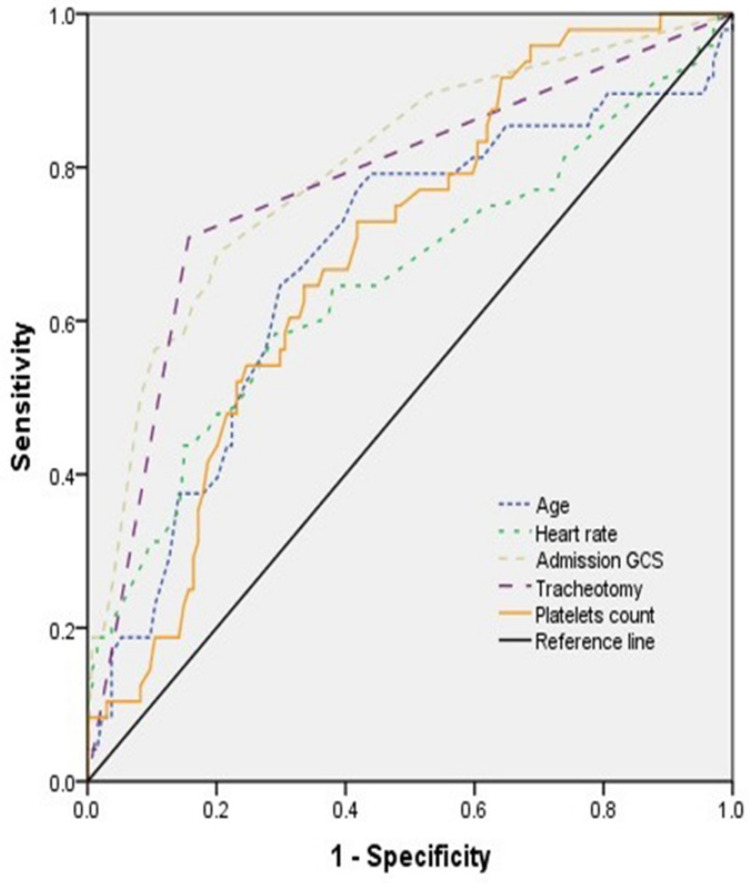
The ROC curve of different variables predicting the prognosis of patients with TBI combined with extracranial trauma.

The ROC curve was used to express sensitivity and specificity of age, admission GCS score, heart rate, tracheotomy, and platelets count for predicting the prognosis (**Figure 2**). The results showed that the area under curve (AUC) of age, admission GCS, heart rate, tracheotomy, and platelets count was 0.678 (95% CI, 0.584-0.771), 0.799 (95% CI, 0.723-0.875), 0.652 (95% CI, 0.553-0.751), 0.776 (95% CI, 0.692-0.859), and 0.688 (95% CI, 0.606-0.770), respectively (**Table 4**).

**Figure 3:**
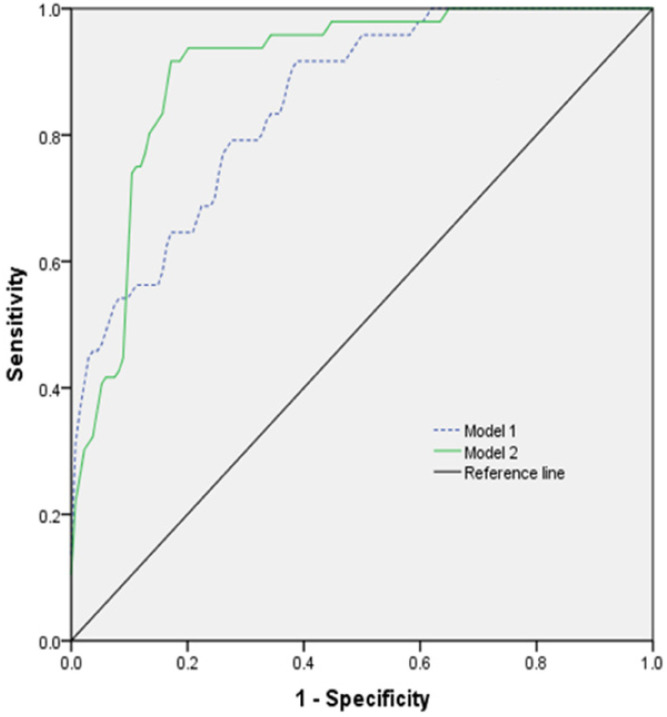
The ROC curve of different models predicting the prognosis of patients with TBI combined with extracranial trauma.

The prognostic model was established by using ROC for multi-factor diagnosis analysis. Model 1 included age, GCS, heart rate, and platelets count; model 2 included age, GCS, heart rate, platelets count, and tracheotomy (**Figure 3**). The AUC of the model 1 was 0.851 (0.792-0.909) with 91.7% sensitivity and 61.9% specificity. The AUC of the model 2 was 0.903 (0.857-0.949) with 91.7% sensitivity and 83.5% specificity (**Table 5**).

## Discussion

This study showed the predictive value of blood routine tests at admission and clinical characteristics for neurological functional outcome in patients with TBI combined with extracranial trauma at one month after discharge in level I trauma center. The purpose of this study was to analyze the clinical characteristics and routine blood test results that may predict the prognosis in patients with TBI combined with extracranial trauma. Moreover, although some blood parameters were predictive in univariate analysis, they did not remain significant after multivariate correction. Therefore, our study focused on the possibility to use blood values at admission and clinical characteristics as prognostic markers in patients with TBI combined with extracranial trauma.

In terms of clinical characteristics, our logistic regression model showed that for each additional year and point of age and heart rate, the risk of unfavorable prognosis increased by 7% and 3.5%, respectively. For each point reduction in initial GCS and platelets at admission within 24 hours after injury, the risk of unfavorable outcome increased by 19.3% and 1.8%, respectively. In addition, anyone undergoing tracheotomy had 15.201-fold higher risk for poor prognosis. The AUC of age, admission GCS score, heart rate, tracheotomy, and platelets count was 0.678, 0.799, 0.652, 0.776, and 0.688, respectively.

Our study indicated a significant correlation between age and outcome at 1-month after discharge in patients with TBI and extracranial trauma. The age distribution histogram revealed that the age of patients was mainly between 40 and 70 years. In the unfavorable group, there were 25% of individuals with older age compared with 11.2% in the favorable group. Older age has been linked to worse outcomes, although possible reasons are still under discussion 32, 33. Among TBI patients, older patients more commonly use anticoagulants and antiplatelet agents 34, 35. Older individuals are more likely to experience domestic falls, which is associated with worse outcome compared with young individuals 1. Moreover, we found that admission GCS score was an independent predictor of prognosis in TBI patients with extracranial trauma, which is consistent with some previous studies in TBI patients 3, 7, 36. We did not confirm the ability of ISS to predict the prognosis of TBI patients after correcting for multiple blood indicators in a multivariate logistic regression model. However, our study confirmed that age and admission GCS can be used as remarkable predictors of the prognosis in TBI patients with extracranial trauma.

Heart rate is a vital sign and could be influenced by some pathological states, such as pain, shock, and intracranial hypertension. Reverse shock index, systolic blood pressure lower than the heart rate, also indicated an unfavorable outcome in patients with severe isolated TBI 37. Moreover, another study revealed that cardio-cerebral network imbalance might influence the relationship of mean arterial pressure, intracranial pressure, and heart rate in sTBI patients 38. As our study showed, heart rate was also an independent factor predicting the outcome in our patient population.

Tracheotomy is a common clinical procedure in patients with sTBI, which provides a stable and tolerated airway to ensure oxygen supply, despite some complications that may accompany the procedure 39. Here we found that tracheotomy was an independent factor for unfavorable outcome. Some studies have shown increased survival in patients with tracheostomy compared with patients who remained intubated after severe TBI 40, 41. However, the optimal time to perform tracheostomy remains a highly controversial topic. Early tracheostomy within 72 hours of admission reduced the duration of mechanical ventilation and length of stay in intensive care unit (ICU) in 120 patients with TBI 42. A randomized trial and a retrospective meta-analysis indicated that early tracheostomy increased risk for hospital death and did not decrease ventilator-associated pneumonia rates 39, 43. In contrast, several studies reported that early tracheostomy could improve prognosis in TBI patients 4.

The majority of patients had normal platelet count, and 29.7% of patients had low platelets in this study. We found that the platelets count was the only significant blood parameter in multivariate logistic regression model, apart from age, admission GCS score, and tracheotomy. Acute coagulopathy of trauma (ACT) is caused by tissue injury and tissue perfusion 44. Coagulopathy is defined as low platelet count or elevated INR or prolonged APTT 45. The platelet dysfunction, increased level of platelet distribution width (PDW), and low platelet count were associated with unfavorable outcome in TBI patients 46-49. The fresh frozen plasma (FFP) resuscitation attenuated platelet dysfunction and improved survival in animals with multiple trauma 50. Furthermore, the importance of ACT in TBI patients has been increasingly recognized. In a large population of patients with TBI, INR and APTT were recognized as independently related to in-hospital mortality 7, 51. High PT was also recognized as a predictor of mortality in patients with trauma 49, 52. However, PT, INR, and APTT were not independent predictors in this study.

Inflammation also plays an important role in TBI and multiple trauma 53, 54. Mass release of proinflammatory factors caused by trauma stimulation or tissue necrosis can lead to leukocyte activation and lymphocytes deficiency 55-57. Neutrophil activation has dual effects on TBI, which might contribute to repair mechanisms or aggravate the pathophysiology of trauma 58, 59. The neutrophil-to-lymphocyte ratio (NLR) is associated with unfavorable outcomes in sTBI patients 8. The majority of our patients had higher leukocyte and neutrophil counts and lower lymphocytes. The inflammatory stimulation caused by multiple injuries may cover the inflammatory manifestations caused by isolated traumatic brain injury, which may explain why inflammatory cell prediction was not significant.

Hypernatremia and hyperglycemia were associated with poor outcome after severe TBI 49, 60-64. The main point is that stress response activates the hypothalamic-pituitary-adrenal axis and sympathetic nervous system, leading to elevated levels of neurohormones and insulin resistance 65. Intensive insulin therapy was considered as a therapeutic strategy to treat cerebral metabolic distress in a previous study 66. However, to prevent hypoglycemia, strict blood sugar control was not recommended 67. Nevertheless, we did not find that high Na+ concentration, hyperglycemia, and high BUN were independent predictors of outcomes in our study. In addition, prognosis of patients with TBI was worse in patients with anemia 49, 68, 69. Multiple trauma was also related with injury-associated anemia 70. More than 50% of the patients in our study population had low hemoglobin levels. However, hemoglobin level was not an independent predictor of neurological prognosis in this study.

We applied the ROC curve and different combination models to evaluate the accuracy of some variables for predicting the outcome in patients with TBI combined with extracranial trauma. Our results showed that total combination models displayed proper accuracy to assess prognosis of patients, with 91.7% sensitivity and 83.5% specificity. The AUC of the model 2 was 0.903 and Youden index was 0.752. The model 2 is useful for predicting the clinical outcome because these variables are easy to obtain. To predict the early prognosis of patients, we excluded tracheotomy as a variable and established the model 1. The AUC of the model 1 was 0.851 and Youden index was 0.536, with 91.7% sensitivity and 61.9% specificity. The AUC of the model 2 was the largest, indicating the highest prognostic accuracy.

This study had several limitations. First, this was a single-center retrospective study, which is why selection bias may have existed. Second, this study investigated a relatively small cohort of patients that may not be significantly representative of a TBI population with severe multiple injuries. Some patient data about pre-injury drug use and more comorbidities were incomplete. In addition, the effects of different parts of multiple injuries on TBI may be different. Finally, the prognosis was assessed with a short-term outcome. Therefore, further randomized studies based on large populations and appropriate follow-up times are needed to provide stronger evidence for predicting patient outcomes.

## Conclusion

We further confirmed that age, admission GCS score, pupillary reflex, tracheotomy, and platelets count can be used as independent predictors of clinical prognosis in patients with severe TBI with extracranial trauma.

## Figures and Tables

**Table 1:** Clinical characteristics of patients in univariate analysis

Characteristic	Total (N=182)	Favorable outcome (N=134)	Unfavorable outcome (N=48)	*p* value
Age (years), mean (SD)	50.41 ± 14.42	48.40 ± 13.42	56.04 ± 15.71	<0.01
Old men (≥65), n (%)	27 (14.8%)	15 (11.2%)	12 (25.0%)	<0.05
Sex (men), n (%)	135 (74.2%)	99 (73.9%)	36 (75.0%)	0.879
Interval time (hours), mean (SD)	10.17 ± 5.77	10.29 ± 5.76	9.83 ± 5.84	0.641
**Injury mechanisms, n (%)**				
Traffic accident	118 (64.8%)	83 (61.9%)	35 (72.9%)	0.367
Falls	43 (23.6%)	35 (26.9%)	7 (14.6%)	
Assaults	5 (2.7%)	4 (3.0%)	1 (2.1%)	
Others/unknown	16 (8.8%)	11 (8.2%)	5 (10.4%)	
Admission GCS score	11.68 ± 3.98	12.87 ± 3.11	8.35 ± 4.26	<0.001
GCS score ≤8, n (%)	48 (26.4%)	20 (14.9%)	28 (58.3%)	<0.001
**Pupil size, n (%)**				
Diameter of both pupils ≥4 mm	7 (3.8%)	1 (0.7%)	6 (12.5%)	<0.001
Diameter of one pupil ≥4 mm	13 (7.1%)	6 (4.5%)	7 (14.6%)	
Diameter of both pupils <4 mm	162 (89.0%)	127 (94.8%)	35 (72.9%)	
**Pupillary reflex, n (%)**				
Brisk	135 (74.2%)	110 (82.1%)	25 (52.1%)	<0.001
Sluggish	24 (13.2%)	15 (11.2%)	9 (18.8%)	
Non-reactive	23 (12.6%)	9 (6.7%)	14 (29.2%)	
Systolic pressure (mm Hg), mean (SD)	124.28 ± 19.69	124.11 ± 16.31	124.75 ± 27.22	0.879
Heart rate, mean (SD)	89.04 ± 32.43	83.83 ± 14.11	102.60 ± 56.51	<0.05
High heart rate (> 100/min), n (%)	40 (22%)	20 (14.9%)	20 (41.7%)	<0.001
ISS score, mean (SD)	26.17 ± 7.45	25.07 ± 6.69	29.23 ± 8.62	<0.01
ISS score > 25, n (%)	81 (44.5%)	52 (38.8%)	29 (60.4%)	<0.05
Head AIS score, mean (SD)	3.63 ± 0.67	3.50 ± 0.65	4.00 ± 0.62	<0.001
Chest injury, n (%)	140 (76.9%)	108 (80.6%)	32 (66.7%)	<0.05
Abdominal injury, n (%)	64 (35.2%)	47 (35.1%)	17 (35.4%)	0.966
Pelvic injury, n (%)	27 (14.8%)	19 (14.2%)	8 (16.7%)	0.677
Limb injury, n (%)	79 (43.4%)	65 (48.5%)	14 (29.2%)	<0.05
Epidural hematoma, n (%)	69 (37.9%)	52 (38.8%)	17 (35.4%)	0.678
Subdural hematoma, n (%)	111 (61.0%)	80 (59.7%)	31 (64.6%)	0.552
Subarachnoid hemorrhage, n (%)	125 (68.7%)	87 (64.9%)	38 (79.2%)	0.068
Cerebral hemorrhage, n (%)	76 (41.8%)	51 (38.1%)	25 (52.1%)	0.091
Skull fracture, n (%)	109 (59.9%)	87 (64.9%)	22 (45.8%)	0.021
Craniotomy, n (%)	32 (17.6%)	15 (11.2%)	17 (35.4%)	<0.01
Tracheal intubation, n (%)	60 (33.0%)	25 (18.7%)	35 (72.9%)	<0.001
Tracheotomy, n (%)	55 (30.2%)	21 (15.7%)	34 (70.8%)	<0.001
Pulmonary infection, n (%)	31 (17.0%)	15 (11.2%)	16 (33.3%)	<0.001
LEVT, n (%)	30 (16.5%)	25 (18.7%)	5 (10.4%)	0.187
Death, n (%)	25 (13.7%)	0	25 (52.1%)	<0.001

GCS: Glasgow Coma Scale; ISS: Injury Severity Score; AIS: Abbreviated injury scale; LEVT: Lower extremity venous thrombosis.

**Table 2:** Routine laboratory parameters of the patients in univariate analysis

Parameters	Total (N=182)	Favorable outcome (N=134)	Unfavorable outcome (N=48)	*p* value
WBC count (×10^9^/L), mean (SD)	12.99 ± 4.33	12.71 ± 4.27	13.79 ± 4.44	0.14
High WBC count, n (%)	138 (75.8%)	100 (74.6%)	38 (79.2%)	0.528
Neutrophils count (×10^9^/L), mean (SD)	11.43 ± 4.09	11.17 ± 4.10	12.14 ± 4.02	0.16
Neutrophils percentage (%), mean (SD)	87.16 ± 5.47	86.95 ± 5.65	87.72 ± 4.93	0.402
High neutrophils percentage, n (%)	175 (96.2%)	128 (95.5%)	47 (97.9%)	0.762
Lymphocytes count (×10^9^/L), mean (SD)	0.80 ± 0.51	0.81 ± 0.53	0.80 ± 0.43	0.887
Lymphocytes percentage (%), mean (SD)	6.67 ± 4.21	6.84 ± 4.31	6.19 ± 3.91	0.357
Low lymphocytes percentage, n (%)	178 (97.8%)	131 (97.8%)	47 (97.9%)	1.000
NLR, mean (SD)	17.85 ± 10.44	17.57 ± 10.73	18.64 ± 9.67	0.544
Platelets count (×10^9^/L), mean (SD)	154.99 ± 53.83	164.10 ± 54.97	129.56 ± 41.36	<0.001
Low platelets count, n (%)	54 (29.7%)	31 (23.1%)	23 (47.9%)	<0.01
Hemoglobin (g/L), mean (SD)	110.90 ± 25.36	113.50 ± 24.09	103.65 ± 27.58	<0.05
Anemia, n (%)	74 (40.7%)	47 (35.1%)	27 (56.3%)	<0.05
Albumin (g/L), mean (SD)	35.43 ± 7.14	36.30 ± 6.33	32.99 ± 8.66	<0.05
Low albumin, n (%)	78 (42.9%)	46 (34.3%)	32 (66.7%)	<0.001
Blood creatinine (µmol/L), mean (SD)	78.96 ± 68.26	70.40 ± 20.20	102.85 ± 126.48	0.083
High creatinine, n (%)	12 (6.6%)	5 (3.7%)	7 (14.6%)	<0.01
Blood Na+ (mmol/L), mean (SD)	141.08 ± 3.57	140.63 ± 3.21	142.34 ± 4.19	<0.05
High Na+ concentration, n (%)	22 (12.1%)	10 (7.5%)	12 (25.0%)	<0.05
Blood K+ (mmol/L), mean (SD)	4.21 ± 0.53	4.17 ± 0.47	4.33 ± 0.67	0.088
Low K+ concentration, n (%)	8 (4.4%)	6 (4.5%)	2 (4.2%)	0.928
Blood BUN (mmol/L), mean (SD)	5.82 ± 2.73	5.50 ± 1.78	6.70 ± 4.32	0.069
High BUN, n (%)	16 (8.8%)	7 (5.2%)	9 (18.8%)	<0.01
Blood UA (µmol/L), mean (SD)	300.12 ± 100.48	295.11 ± 95.41	314.09 ± 113.37	0.304
High UA, n (%)	26 (14.3%)	15 (11.2%)	11 (22.9%)	<0.05
Blood glucose (mmol/L), mean (SD)	8.32 ± 2.78	8.02 ± 2.52	9.14 ± 3.28	<0.05
High glucose, n (%)	87 (47.8%)	57 (42.5%)	30 (62.5%)	<0.05
PT (s), mean (SD)	15.64 ± 2.78	15.15 ± 1.67	16.98 ± 4.40	<0.01
High PT, n (%)	96 (52.7%)	59 (44.0%)	37 (77.1%)	<0.001
INR, mean (SD)	1.60 ± 4.59	1.67 ± 5.34	1.40 ± 0.49	0.733
High INR, n (%)	79 (43.4%)	48 (35.8%)	31 (64.6%)	<0.01
Fibrinogen (g/L), mean (SD)	2.35 ± 1.01	2.41 ± 1.06	2.17 ± 0.87	0.149
Low fibrinogen, n (%)	65 (35.7%)	42 (31.3%)	23 (47.9%)	<0.05
APTT (s), mean (SD)	37.45 ± 7.44	36.38 ± 4.69	40.41 ± 11.77	<0.05
High APTT, n (%)	16 (8.8%)	7 (5.2%)	9 (18.8%)	<0.01
TT (s), mean (SD)	16.15 ± 2.01	15.92 ± 1.52	16.80 ± 2.91	0.051
High TT, n (%)	13 (7.1%)	7 (5.2%)	6 (12.5%)	0.093

WBC: White blood cells; NLR: Neutrophil lymphocyte ratio; BUN: Blood urea nitrogen; UA: Uric acid; PT: Prothrombin time; INR: International normalized ratio; APTT: Activated partial thromboplastin time; TT: Thrombin time.

**Table 3:** Clinical outcomes in the patient population in multivariate regression analysis

Variable	OR	95% CI	ρ value	B
Age	1.070	1.018-1.124	**0.007**	0.068
Admission GCS score	0.807	0.675-0.965	**0.019**	-0.215
Pupil size	0.224	0.049-1.023	0.054	-1.498
Pupillary reflex	0.595	0.185-1.915	0.384	-0.520
Heart rate	1.035	1.004-1.067	**0.028**	0.034
Admission ISS score	0.970	0.888-1.060	0.504	-0.030
Head AIS score	1.326	0.497-3.539	0.573	0.282
Craniotomy	1.441	0.381-5.447	0.590	0.365
Tracheal intubation	0.923	0.241-3.538	0.907	-0.080
Tracheotomy	15.201	4.121-56.078	**0.000**	2.721
Platelets count	0.982	0.967-0.997	**0.022**	-0.018
Hemoglobin	0.987	0.958-1.016	0.367	-0.014
Blood albumin	1.137	0.990-1.304	0.068	0.128
Blood creatinine	1.012	0.993-1.031	0.218	0.012
Blood Na+ concentration	0.960	0.801-1.152	0.664	-0.040
Blood K+ concentration	0.718	0.234-2.201	0.562	-0.331
Blood BUN	0.775	0.553-1.086	0.139	-0.255
Blood glucose	0.959	0.775-1.188	0.705	-0.041
PT	1.060	0.644-1.745	0.819	0.058
APTT	0.996	0.871-1.139	0.956	-0.004
TT	0.994	0.649-1.520	0.976	-0.006

GCS: Glasgow Coma Scale; ISS: Injury Severity Score; AIS: Abbreviated injury scale; BUN: Blood urea nitrogen; PT: Prothrombin time; APTT: Activated partial thromboplastin time; TT: Thrombin time.

**Table 4:** Results of work characteristic of AUC with different variables predicting prognosis one month after discharge

Variable	Youden index	Sensitivity	Specificity	AUC	95% CI	*p* value
Age	0.353	0.771	0.582	0.678	0.584-0.771	<0.001
Admission GCS	0.486	0.688	0.798	0.799	0.723-0.875	<0.001
Heart rate	0.292	0.583	0.709	0.652	0.553-0.751	<0.05
Tracheotomy	0.552	0.708	0.843	0.776	0.692-0.859	<0.001
Platelets count	0.311	0.729	0.582	0.688	0.606-0.770	<0.001

GCS: Glasgow Coma Scale; AUC: Area Under Curve.

**Table 5:** Results of work characteristic of AUC with different models predicting prognosis one month after discharge

Model	Youden index	Sensitivity	Specificity	AUC	95% CI	*p* value
Model 1	0.536	0.917	0.619	0.851	0.792-0.909	<0.001
Model 2	0.752	0.917	0.835	0.903	0.857-0.949	<0.001

Model 1: Age + GCS + Heart rate + Platelets count; Model 2: Age + GCS + Heart rate + Platelets count + Tracheotomy. AUC: Area Under Curve.

## Abbreviations

sTBI: severe traumatic brain injury
CI: confidence interval
ORs: odds ratios
SD: standard deviation
GCS: Glasgow Coma Scale
GOSE: Glasgow Outcome Scale Extended
ISS: Injury Severity Score
AIS: abbreviated injury scale
LEVT: lower extremity venous thrombosis
WBC: white blood cell
NLR: neutrophil lymphocyte ratio
BUN: blood urea nitrogen
UA: uric acid
PT: prothrombin time
APTT: activated partial thromboplastin time
INR: International Normalized Ratio
TT: thrombin time
